# Danggui Beimu Kushen Pill Alleviates Colitis-Induced Inflammation in Mice by Regulating the IL-6/IL-6R and IL-17A/IL-17RA Signaling Pathways

**DOI:** 10.3390/ph18020141

**Published:** 2025-01-22

**Authors:** Shuo Gong, Ran Xu, Yuqing Wang, Shangling Mao, Yi Zhang, Qingru Bu, Ruotong Yang, Tianming Wang, Yue Yang

**Affiliations:** 1 School of Integrated Chinese and Western Medicine, Anhui University of Chinese Medicine, 350 Longzihu Road, Hefei 230012, China; gongshuo0221@163.com (S.G.); 13395621206@163.com (R.X.); wangyuqing202301@163.com (Y.W.); maoshangling0717@163.com (S.M.); buqingru0703@163.com (Q.B.); yangrt99@163.com (R.Y.); 2 School of Chinese Medicine, Bozhou University, 2266 TangwangAve., Bozhou 236800, China; zhangyi20201125@163.com

**Keywords:** Danggui Beimu Kushen pill, ulcerative colitis, interleukin-6, interleukin-17, Th17

## Abstract

**Background/Objectives**: Danggui Beimu Kushen pill (DBK) is a traditional Chinese medicine renowned for its efficacy in the treatment of inflammatory conditions. It has been used in the modern clinical treatment of ulcerative colitis (UC). Further research is required to clarify its underlying mechanisms. **Methods**: DBK compounds were analyzed using UPLC-ESI-MS/MS. A mouse model of Dextran sulfate sodium (DSS)-induced UC was used to assess the efficacy of DBK. Network pharmacology identified DBK targets in UC, which were validated by molecular docking simulations. Cytokine levels were quantified using ELISA. Western blotting, immunofluorescence (IF), and immunohistochemistry (IHC) were employed to explore its underlying molecular mechanisms. **Results**: DBK treatment enhanced body weight and colon length in mice with DSS-induced colitis. Inflammatory cytokine levels (TNF-α, IL-1β, IL-6, IL-23, and IL-17A) were notably decreased in both serum and colon tissues. Network pharmacology and molecular docking analyses identified the IL-6/IL-6R and IL-17A/IL-17RA signaling pathways as crucial for the DBK treatment of UC. Additional validation using WB, IHC, and IF analyses demonstrated that DBK alleviated UC by suppressing the IL-6/STAT3 and IL-17/TRAF6/NF-κB pathways. **Conclusions**: DBK mitigates intestinal injury in mice with DSS-induced colitis and exerts therapeutic effects on UC by inhibiting the IL-6/IL-6R and IL-17A/IL-17RA signaling pathways to reduce inflammation. These findings provide significant insights into the mechanism of DBK treatment for UC.

## 1. Introduction

Ulcerative colitis (UC) is a persistent inflammatory disorder characterized by extended periods and frequent relapse. Clinical symptoms encompass persistent mucosal inflammation of the rectum and colon accompanied by diarrhea, abdominal pain, rectal bleeding, and weight loss [1]. The treatment of UC primarily involves medications such as aminosalicylates, glucocorticoids, corticosteroids, and immunosuppressants. However, these drugs have notable side effects, limited tolerance, and potential dependency, making their long-term use problematic [1,2]. Although scientific progress has introduced advanced therapies in clinical settings, medication failure rates remain high, necessitating surgery in over 10% of patients [3]. Consequently, it is crucial to investigate novel treatments and approaches to mitigate gastrointestinal symptoms in patients with UC.

Dysregulated immune responses are integral to the multifactorial pathogenesis of UC. Interleukin-6 (IL-6) is a versatile cytokine linked to persistent inflammatory responses [4]. Increased secretion of IL-6 exhibits a direct positive correlation with the magnitude of the inflammatory response [5]. IL-6 interacts with membrane-bound or soluble IL-6 receptors to form a complex that recruits gp130 to the cell surface to activate intracellular Janus kinase (JAK) [6]. Subsequently, activated JAK phosphorylates STAT3 (signal transducer and activator of transcription 3), leading to the formation of STAT3 dimers and their translocation into the nucleus [7]. Within the nucleus, STAT3 dimers recognize and bind to specific promoter regions of IL-6, thereby initiating the transcriptional process of IL-6 [8].

Maintaining intestinal stability relies on the balance between Th17 and Treg cells. In patients with UC, this equilibrium is disrupted, manifesting an increase in Th17 cells and/or impaired Treg cells [9]. Th17 cells predominantly release IL-17A, which stimulates chemokine production, attracts neutrophils and other immune cells to inflammatory sites, and intensifies the inflammatory response. TRAF6 serves as a critical adaptor protein in the IL-17 receptor signaling pathway, activating downstream NF-κB pathways and promoting the transcription of the pro-inflammatory cytokine IL-6 [10]. The IL-6/IL-6R and IL-17A/IL-17RA signaling pathways both enhance the inflammatory response. IL-6 and IL-17 inhibitors have recently been identified as promising biological agents for the treatment of inflammatory bowel diseases (IBDs), including UC, by reducing intestinal inflammation and enhancing patient quality of life [11].

UC treatment requires individualized therapy based on the patient’s specific syndrome type and disease severity. The therapeutic benefits of traditional Chinese medicine (TCM) for UC are rooted in its comprehensive approach to holistic adjustment and personalized treatment strategies. TCM focuses on modulating a patient’s physiological state and enhancing the intestinal milieu, thereby achieving symptom mitigation and the minimization of recurrence [12]. According to TCM principles, the pathogenesis of UC is primarily associated with the accumulation of dampness and heat, which manifest clinically as bloody stools. In TCM, the therapeutic strategy to “clear heat and cool blood” aims to reduce inflammation and hemostasis. This does not directly equate to antipyretic actions but rather refers to the alleviation of inflammatory responses and the normalization of blood circulation to prevent bleeding. For a more precise understanding, this approach can be interpreted as targeting the reduction of pro-inflammatory cytokines and the modulation of vascular integrity [7]. The Danggui Beimu Kushen pill (DBK), formulated by Zhongjing Zhang during the Han Dynasty, is recorded in the *Jin Gui Yao Lue*. DBK is composed of three herbs, namely, *Angelica sinensis* (Oliv.) Diels (Danggui, Chinese), *Fritillaria thunbergii* Miq. (Beimu, Chinese), and *Sophora flavescens* Aiton (Kushen, Chinese), in a ratio of 1:1:1. DBK has been used in previous research against different diseases, including UC, cancers, and chronic prostatitis [13]. Danggui is recognized for its ability to enrich the blood, enhance circulation, and relieve pain (Chinese). It contains active ingredients like ferulic acid and polysaccharides that enhance hematopoiesis by stimulating stem cell proliferation and differentiation, thereby increasing red blood cell quantity and quality [14]. It also improves blood circulation by inhibiting platelet aggregation, reducing viscosity, and dilating blood vessels [15]. Additionally, it has analgesic effects by reducing inflammation [16]. Beimu possesses the efficacy of clearing heat and detoxification. Kushen has the function of clearing heat and dehumidification, and it can be used in the treatment of dysentery with heat and hematochezia. This formula, which is known for its heat-clearing, damp-drying, blood-cooling, and bleeding-stopping properties, is commonly used in the clinical setting to treat UC. However, scientific proof of DBK is still lacking in some cases. This study aims to investigate the potential anti-inflammatory effects of DBK and its underlying mechanisms in a UC mouse model, with the goal of exploring its therapeutic potential for personalized UC treatment.

## 2. Results

### 2.1. Identification and Overview of Compounds in DBK

**Figure 1:**
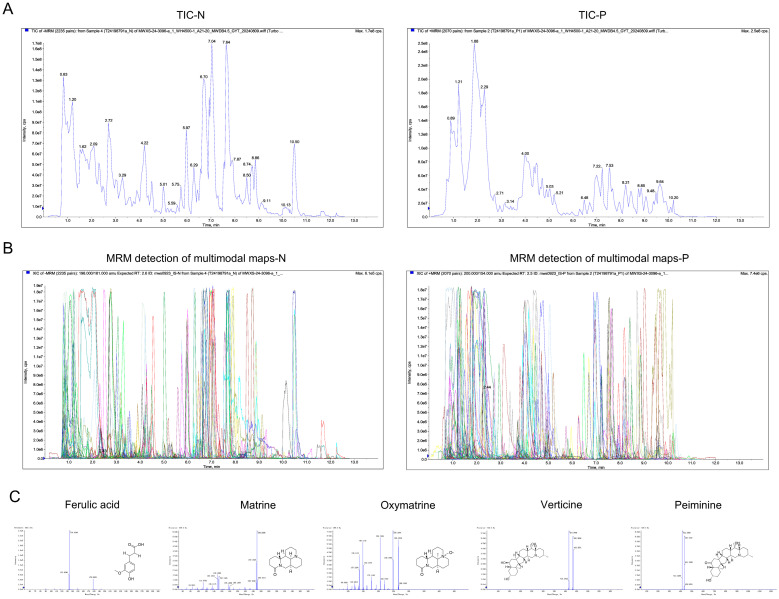
Analysis of DBK components using UPLC-ESI-MS/MS. (**A**) Total ion chromatograms (TICs) of DBK; (**B**) multiple reaction monitoring (MRM) detections for multimodal maps of DBK, the different colors typically represent different ion pairs or compounds; (**C**) the secondary mass spectrum of the principal components of DBK (ferulic acid, matrine, oxymatrine, verticine, and peiminine). cps: count per second.

UPLC-ESI-MS/MS was conducted to thoroughly identify the components of DBK. The total ion current (TIC) and multiple reaction monitoring (MRM) detections for multimodal maps of the DBK samples are presented in Figure 1A,B. In DBK, 816 compounds were identified, including 189 flavonoids, 152 lipids, 86 alkaloids, 83 phenolic acids, 67 amino acids and derivatives, 52 lignans and coumarins, 31 terpenoids, 28 organic acids, 15 nucleotides and derivatives, 8 quinones, and 105 other compounds. The secondary mass spectrum of the principal components of DBK (ferulic acid, matrine, oxymatrine, verticine, and peiminine) obtained using UPLC-ESI-MS/MS is shown in Figure 1C and Table 1.

### 2.2. DBK Reduces Intestinal Damage Caused by DSS in Mice

**Figure 2:**
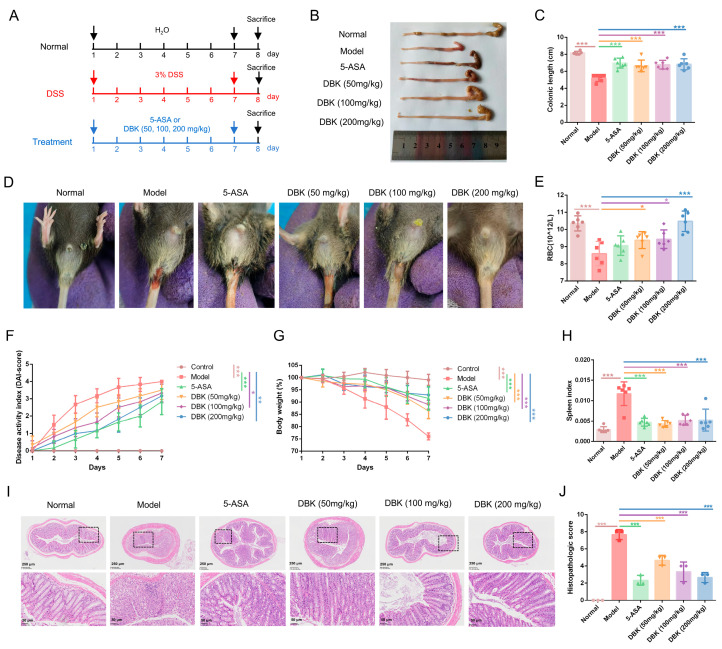
DBK reduced DSS-induced intestinal damage in mice. (**A**) Establishment of a DSS-induced colitis mouse model and administration of DBK; (**B**) length of colons; (**C**) colon length analysis; (**D**) stool bleeding; (**E**) red blood cell (RBC) counts in peripheral blood; (**F**) DAI scores; (**G**) changes in body weight; (**H**) spleen index; (**I**) H&E staining of colonic tissue, the black dashed boxes indicate areas of interest magnified in the bottom row; (**J**) histological scores. Data are expressed as mean ± SD (*n* = 6). * *p* < 0.05; ** *p* < 0.01; *** *p* < 0.001.

A DSS-induced colitis mouse model was developed to evaluate DBK’s therapeutic potential in treating UC (Figure 2A). The colons of the mice in the model group were shortened after seven days of casting and dosing. The groups treated with 5-aminosalicylic acid (5-ASA) and DBK showed a noticeable improvement in colon length (Figure 2B,C). Mice treated with DSS exhibited symptoms of diarrhea and bloody stools (Figure 2D), as well as a decrease in the peripheral blood RBC (red blood cells) ratio (Figure 2E). DBK improved these conditions, particularly at a dose of 200 mg/kg, with effects better than those of 5-ASA. The disease activity index (DAI) scores were reduced in both the 5-ASA and DBK treatment groups compared with those in the model group, with the DBK 200 mg/kg group showing the greatest reduction (Figure 2F). Compared to normal mice, those in the model group started to exhibit weight loss beginning on the fourth day. Following DBK treatment, the mice exhibited a significant increase in body weight relative to the model group (Figure 2G). The spleen index, an important indicator of immune function, improved significantly after intervention with DBK and 5-ASA (Figure 2H). Consistently, the histopathological examination of colon tissues revealed that the mice in the model group exhibited severe pathological alterations, characterized by a marked loss of goblet and epithelial cells, pronounced inflammatory cell infiltration, and extensive damage to crypt structures. In contrast, intestinal epithelial integrity was preserved in mice treated with DBK and 5-ASA, demonstrating a significant reduction in crypt architectural disruption and inflammatory cell infiltration (Figure 2I,J).

**Figure 3:**
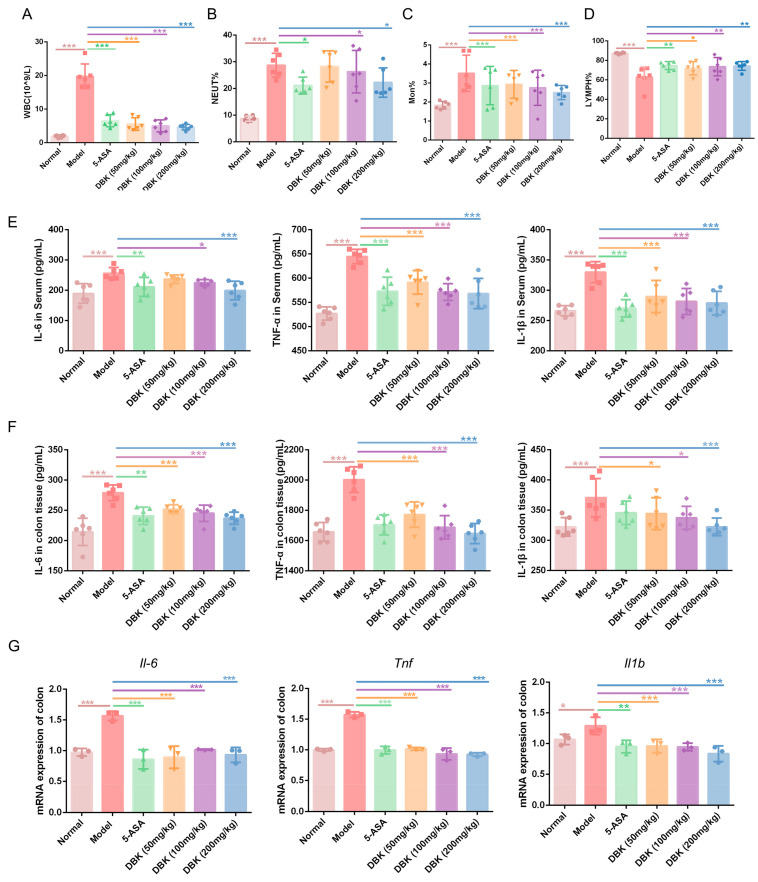
DBK regulated inflammatory cells and factors. (**A**) Changes in white blood cell number (WBC); (**B**) cell number of neutrophils (NEU); (**C**) cell number of monocytes (Mon); (**D**) cell number of lymphocytes (LYMPH); (**E**) concentration of IL-6, TNF-α, and IL-1β in serum. (**F**) concentration of IL-6, TNF-α, and IL-1β in tissues; (**G**) mRNA expression of *Il-6*, *Tnf*, and *Il1b* in colon tissues. Data are expressed as mean ± SD (*n* = 6). * *p* < 0.05; ** *p* < 0.01; *** *p* < 0.001.

Peripheral blood monocyte (Mon), lymphocyte (LYMPH), neutrophil (Neu), and white blood cell (WBC) levels are used as inflammatory markers in patients with UC. In the model group of mice, there was a significant increase in inflammatory cells infiltrating the body, resulting in significant increases in WBC, Neu, and Mon levels. The three DBK doses (50, 100, and 200 mg/kg) demonstrated comparable efficacy to the positive control drug 5-ASA in decreasing WBC proportions (Figure 3A), Neu (Figure 3B), and Mon (Figure 3C) in the peripheral blood of UC mice. In addition, 5-ASA and DBK restored the proportion of LYMPH (Figure 3D). The ELISA results showed a notable reduction in the expression levels of the pro-inflammatory factors IL-6, TNF-α, and IL-1β in the serum and tissues of treated mice compared to the model group (Figure 3E,F). In alignment with the ELISA findings, quantitative polymerase chain reaction (qPCR) experiments demonstrated that DSS elevated Il-6, Tnf, and Il1b mRNA expression levels, while DBK treatment counteracted these effects (Figure 3G). These findings indicate that DBK treatment led to a marked increase in colon length, reduced rectal bleeding, and a significant reduction in inflammatory cell infiltration within the colon. In brief, the overall health status of the mice with ulcerative colitis was notably improved, and the inflammatory symptoms were effectively alleviated.

### 2.3. Potential Mechanism of DBK Identified by Network Pharmacology and Molecular Docking

**Figure 4:**
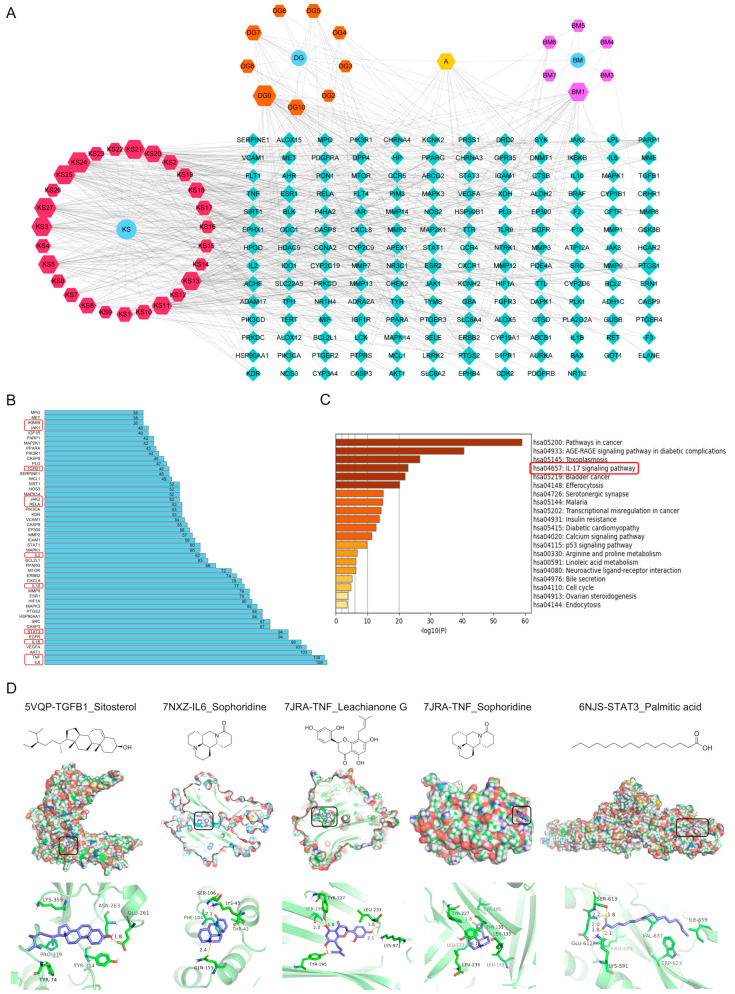
Network pharmacology and molecular docking results. (**A**) Construction of a “DBK-therapeutic ingredient target” network using Cytoscape software (DG: Danggui, BM: Beimu, KS: Kushen); (**B**) top 50 core targets identified for DBK therapy in the management of UC; (**C**) KEGG enrichment analysis; (**D**) interaction of TGFB1–sitosterol, IL6–sophoridine, TNF–leachianone G, TNF–sophoridine, and STAT3–palmitic acid.

To uncover the potential signaling pathways regulated by DBK in the treatment of UC and elucidate the relationship between DBK components and their targets, we employed the research methodology of network pharmacology and molecular docking. The active compounds in DBK were screened in the TCMSP database and reports, and 9 compounds from Danggui, 6 from Beimu, and 11 from Kushen were identified (Please refer to Supplementary Table S1 for a list of these 26 compounds). Using the Swiss Target Prediction database, 166 potential targets of the active compounds were identified. The overlapping targets were integrated into Cytoscape (version 3.8.0) to construct the ‘DBK-therapeutic ingredient target’ network (Figure 4A). Moreover, within the top 50 core targets for DBK therapy in the treatment of UC, we identified a range of significant molecules: inflammatory cytokines like TNF, IL10, and IL2; components of the IL-6/JAK/STAT3 signaling pathway, encompassing IL6, JAK1, JAK2, and STAT3; as well as TGFB1 (transforming growth factor beta-1), RELA, and IKBKB, which are integral to the IL-17/TRAF6/NF-κB pathway (Figure 4B). KEGG enrichment analysis revealed that the UC pathway was engaged via the IL-17 signaling pathway (Figure 4C). The IL-6/JAK/STAT3 and IL-17/TRAF6/NFKB signaling pathways are essential for DBK’s effectiveness in treating UC. Molecular docking results demonstrated that sitosterol binds effectively to TGFB1, forming a hydrogen bond with Glu261 and engaging in van der Waals interactions with Tyr74, Tyr314, Pro319, Lys359, and Asn263. Sophoridine effectively binds to IL6, forming two hydrogen bonds with Gln155 and Ser106. Leachianone G effectively binds to TNF, forming five hydrogen bonds with Ler233, Lys87, Tyr227, Ser136, and Tyr195. Upon docking into TNF, sophoridine forms a hydrogen bond with Tyr227 and engages in a π-cation interaction with Tyr135. Palmitic acid binds effectively to STAT3, forming three hydrogen bonds with Ser613 and Ser611 and two salt bridges with Lys591 and Arg609. The calculated docking scores for these compounds were −4.980, −6.693, −14.491, −6.649, and −5.615, respectively (Figure 4D). These findings support further investigation into the therapeutic potential of DBK.

### 2.4. DBK Exerts Anti-Inflammatory Effects by Inhibiting IL-6/STAT3 Pathway

**Figure 5:**
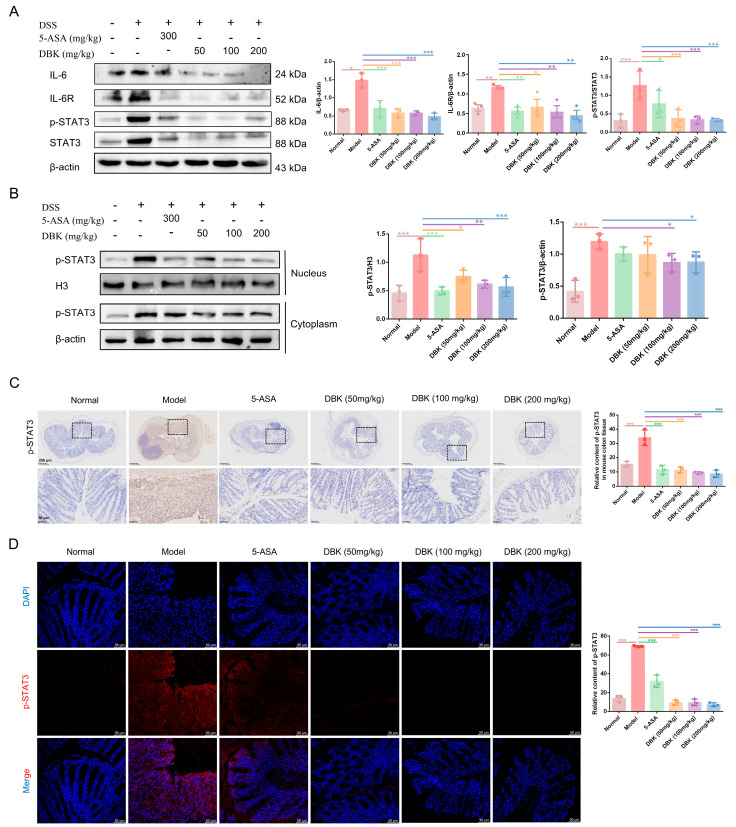
DBK inhibited the IL-6/STAT3 signaling pathway. (**A**) Protein expression of IL-6, IL-6R, p-STAT3, and STAT3; (**B**) protein expression of p-STAT3 in the cytoplasm and nucleus; (**C**) IHC staining of p-STAT3 in the colon of mice in each group, the black dashed boxes indicate areas of interest magnified in the bottom row; (**D**) IF staining of p-STAT3 in the colon. Data are expressed as mean ± SD (*n* = 3). * *p* < 0.05; ** *p* < 0.01; *** *p* < 0.001.

We conducted molecular experiments to validate our hypothesis that DBK modulates the IL-6/JAK/STAT3 pathway. In the UC mouse model, there was a notable increase in the protein expression levels of IL-6, IL-6R, and p-STAT3 compared with those in the normal group, whereas the protein expression exhibited a decrease after DBK treatment in a concentration-dependent manner (Figure 5A). Furthermore, the protein expression of p-STAT3 in the nuclei of the model group was elevated compared to that in the normal group. DBK treatment led to a dose-dependent reduction in nuclear expression levels (Figure 5B). The results of IHC (Figure 5C) and IF (Figure 5D) were consistent with the Western blotting results, suggesting that DBK can decrease the protein expression of p-STAT3. These results collectively indicate that we have further validated STAT3 as a key target in UC, and that DBK inhibits the phosphorylation of STAT3 by modulating IL-6/IL-6R signaling.

### 2.5. Impact of DBK on Th17/Treg Imbalance in UC Mice

**Figure 6:**
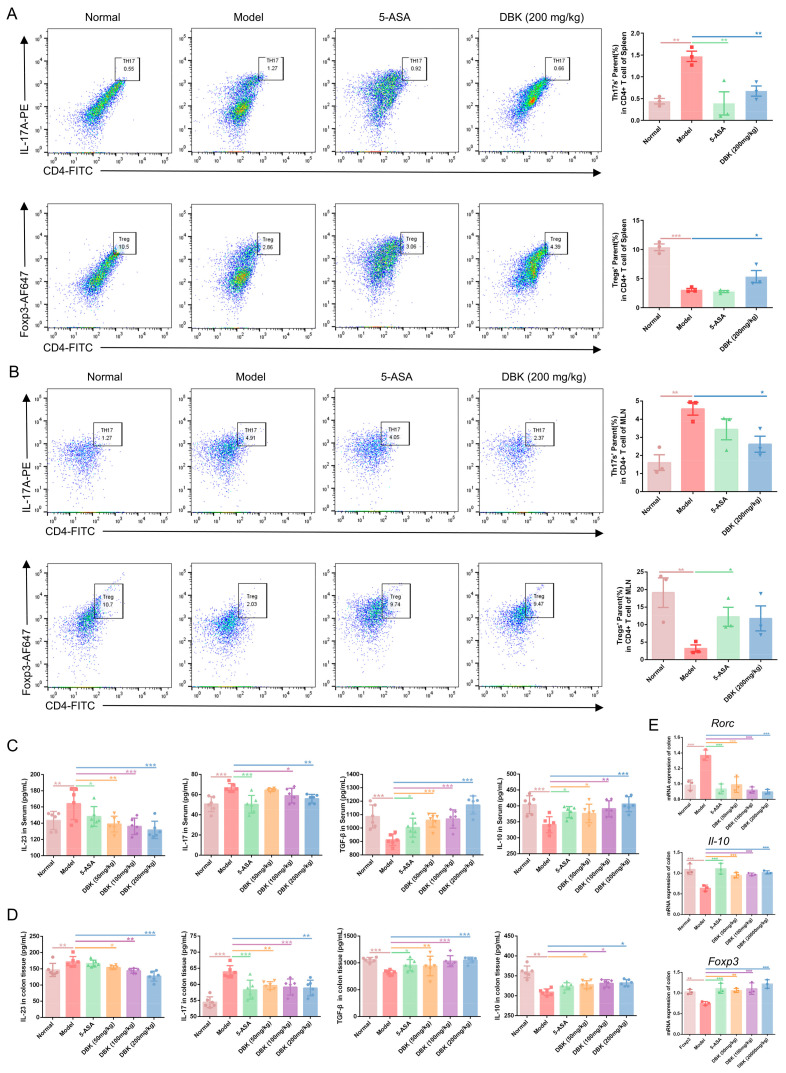
Effect of DBK on Th17/Treg balance in UC mice. (**A**) The proportion of Th17 and Treg cells in the spleen was detected via flow cytometric analysis (*n* = 3); (**B**) the proportion of Th17 and Treg cells in the colonic MLN was detected via flow cytometric analysis (*n* = 3); (**C**) concentration of IL-23, IL-17, TGF-β, and IL-10 in serum (*n* = 6); (**D**) concentration of IL-23, IL-17, TGF-β, and IL-10 in tissues (*n* = 6); (**E**) the mRNA expression of *Rorc*, *Il10*, and *Foxp3* was measured using qPCR (*n* = 3). Data are presented as mean ± SD. * *p* < 0.05; ** *p* < 0.01; *** *p* < 0.001.

In UC, an imbalance between Th17 and Treg cells may worsen intestinal inflammation. Flow cytometry was employed to investigate the impact of DBK on Th17/Treg ratios in the spleen and mesenteric lymph nodes (MLN). DSS treatment increased Th17 cell proportions and decreased Treg cell proportions in the spleen compared to those in the control group; these effects were reversed by DBK and 5-ASA treatment (Figure 6A). Consistent results were observed in MLN, showing that DBK improved the Th17/Treg imbalance (Figure 6B). IL-23 facilitates Th17 cell differentiation, leading to IL-17 secretion. The ELISA results revealed that compared to the model group, treatment with DBK and 5-ASA significantly reduced the levels of the pro-inflammatory factors IL-23 and IL-17A in the mouse serum. Transforming growth factor-β (TGF-β) promotes the differentiation of Tregs, which in turn secrete IL-10. The ELISA results demonstrated a notable reduction in the serum levels of the inhibitory cytokines TGF-β and IL-10 in the model group mice, with improved recovery following 5-ASA and DBK treatment (Figure 6C). The concentrations of these pro-inflammatory and anti-inflammatory factors in colon tissues were consistent with those in the serum (Figure 6D). *RORC* encodes the transcription factor RORγt, which induces IL-17A production in Th17 cells. IL-17A downregulates FOXP3 expression and reduces IL-10 production in Tregs. qPCR analysis indicated a marked increase in RORγt and IL-17A mRNA expression and a significant decrease in IL-10 expression in the colon tissues of mice with UC. Following treatment with 5-ASA and DBK, there was a decrease in the mRNA expression of RORγt and IL-17A, and an increase in IL-10 levels (Figure 6E). This study shows that DBK administration effectively reduces Th17 cell overexpression in UC mice and restores the Th17/Treg cell balance.

### 2.6. DBK Mitigates Inflammation by Suppressing the IL-17/TRAF6/NF-κB Signaling Pathway

**Figure 7:**
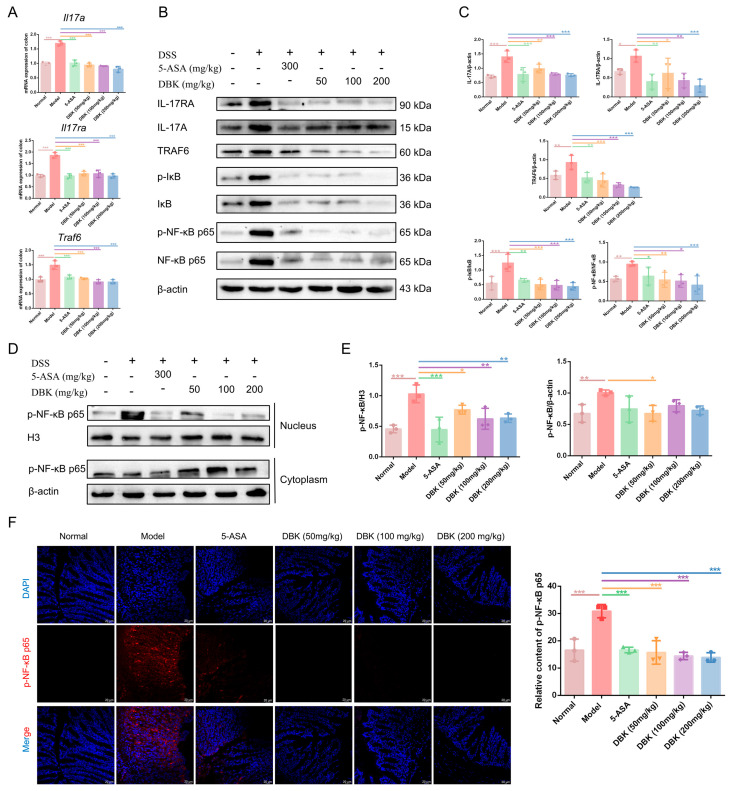
DBK inhibited the IL-17A/TRAF/NF-κB signaling pathway. (**A**) mRNA expression of *Il-17a*, *Il17ra*, and *Traf6* in the colon tissue of each group; (**B**) protein expression of IL-17RA, IL-17, TRAF6, p-IκB, IκB, p-NF-κB p65, and NF-κB p65 in the colon tissue; (**C**) densitometric quantification of the ratio of IL-17A/β-actin, IL-17RA/β-actin, TRAF6/β-actin, p-IκB/IκB, and p-NF-κB p65/NF-κB p65; (**D**) protein expression of p-NF-κB p65 in the cytoplasm and nucleus; (**E**) densitometric quantification of the ratio of p-NF-κB p65/H3 and p-NF-κB p65/β-actin; (**F**) immunofluorescence (IF) staining for p-NF-κB p65 in the colons. Data are expressed as mean ± SD (*n* = 3). * *p* < 0.05; ** *p* < 0.01; *** *p* < 0.001.

To ascertain whether DBK inhibited the increase in Th17 cells and the subsequent increase in IL-17A secretion in the model group, thereby activating the IL-17/TRAF6/NF-κB pathway and leading to the secretion of more inflammatory factors, qPCR, Western blotting, and IF assays were performed. qPCR revealed that DSS induced an increase in IL-17A and TRAF6 mRNA expression, whereas DBK treatment reversed these alterations (Figure 7A). Simultaneously, we observed an increased protein expression of IL-17A, IL-17RA, and TRAF6 in the model group mice. After treatment with DBK, the protein expression levels of the three different dosage groups were reduced. The model mouse group exhibited elevated protein levels of p-IκB and p-NF-κB p65 (Figure 7B,C). The model group exhibited increased nuclear protein expression of p-NF-κB p65 compared to that in the normal group. Following treatment with DBK, the expression levels in the nucleus decreased (Figure 7D,E). IF results indicated elevated p-NF-κB p65 expression in the model group compared to that in the normal group, which significantly decreased following DBK treatment (Figure 7F). These findings suggest that DBK reduces inflammation by targeting the IL-17/TRAF6/NF-κB pathway.

## 3. Discussion

The chronic activation of the intestinal mucosal inflammatory response is central to UC pathology, highlighting the importance of anti-inflammatory treatment. DBK, a well-known traditional Chinese formula recognized for its anti-inflammatory effects, is utilized in clinical settings to treat UC [13]. However, the mechanisms by which DBK treats UC have not been fully elucidated. In this study, we confirmed that DBK alleviated inflammation in mice with colitis. We provide strong evidence that IL-6/IL-6R and IL-17A/IL-17RA signaling are crucial in this process.

IL-6 is a multifunctional pro-inflammatory cytokine that significantly contributes to chronic inflammation and is pivotal in the pathogenesis of UC. In patients with UC, the levels of IL-6 in the serum and intestinal mucosa are elevated. Excessive expression of IL-6 can affect electrolyte secretion in the body and intestinal epithelial cells, ultimately leading to the gradual infiltration of neutrophils at the site of inflammation [11,17]. The IL-6 signaling pathway begins when IL-6 binds to either membrane-bound or soluble IL-6R, subsequently activating the JAK/STAT3 pathway [18]. Aberrantly activated STAT3 is frequently observed in inflammatory diseases, cancer, and autoimmune disorders [19]. Molecules within this signaling axis, such as IL-6, JAK, and STAT3, are critical targets for drug development [20]. In our study, network pharmacology and molecular docking experiments have shown that the core targets, including IL-6, STAT3, JAK1, and JAK2, are closely related to DBK. The compounds in DBK, such as sophoridine, palmitic acid, sitosterol, and leachianone G exhibited good binding activity with IL-6, STAT3, TGF-β, and TNF-α, respectively. Notably, the interaction between IL-6 and STAT3 is bidirectional [21]. After an increase in IL-6 secretion, it rapidly activates the JAK/STAT3 pathway. Activated STAT3 can promote the transcription of IL-6, ultimately amplifying the IL-6 signaling cascade [22]. Elevated STAT3 phosphorylation has been observed in both ulcerative colitis patients and specific animal colitis models. This study showed that DBK alleviated colonic inflammation in UC mice by decreasing IL-6 levels and inhibiting STAT3 expression and phosphorylation in vivo.

IL-17A’s pro-inflammatory properties are essential for host protection, but unchecked IL-17 signaling can lead to immunopathology, autoimmune diseases, and cancer progression [23]. IL-17A affects various downstream cells, inducing the production and release of pro-inflammatory cytokines, chemokines, antimicrobial peptides, and matrix metalloproteinases. This process results in inflammatory cell infiltration and tissue damage, thereby intensifying local inflammatory responses [24]. In UC patients, IL-17A expression is elevated at lesion sites compared to normal mucosa, with significantly higher levels in severe cases than in mild to moderate cases [25]. In our study, IL-17A expression levels in colonic tissue and serum were elevated in mice with DSS-induced UC compared to those in the normal group. The expression level of IL-17A is downregulated by DBK in a concentration-dependent manner. IL-17 signaling is initiated when IL-17 binds to its heterodimeric receptors, such as the IL-17RA and IL-17RC complex. IL-17 signaling begins when it binds to its heterodimeric receptors, such as the IL-17RA and IL-17RC complex. Binding triggers conformational changes in the receptor complex, activating downstream signaling. The adaptor protein Act1 is crucial for facilitating receptor complex binding and recruiting molecules, such as TRAF6. Act1 and TRAF6 activate the NF-κB pathway, resulting in the expression of pro-inflammatory cytokine genes, such as IL-6 and TNF-α [26]. DBK alleviates intestinal inflammation in UC by reducing pro-inflammatory cytokine release, decreasing IL-17 pathway protein expression in the colon, and inhibiting p-NF-κB p65 nuclear entry. The complex regulatory network formed by the interplay of multiple cytokines and the IL-6/JAK/STAT3 and IL-17/TRAF6/NF-κB signaling pathways is crucial for inflammatory processes. IL-6 can intensify inflammation via autocrine and paracrine mechanisms and promote Th17 cell differentiation [11,27,28]. Th17 cells produce the pro-inflammatory cytokine IL-17, whereas Treg cells secrete anti-inflammatory cytokines that play a crucial role in suppressing inflammation and protecting the colonic mucosa from harm [29,30]. Maintaining a balance between Th17 and Treg cells is crucial for maintaining the integrity of the intestinal immune system [31]. Flow cytometry analysis in this study demonstrated that DBK treatment significantly decreased Th17 cell proportions in mice and partially restored Treg cell numbers. IL-6 initiates Th17 cell differentiation, while IL-23 is crucial for the maintenance and proliferation of these cells in later stages [32]. IL-23 enhances IL-17 production by supporting Th17 cell function, thus synergistically promoting inflammatory responses [33]. TGF-β induces naive T cell differentiation into Tregs in the thymus and supports their expansion and stabilization in the periphery. Furthermore, TGF-β directly augments the immunosuppressive capabilities of Tregs by suppressing effector T-cell activation and proliferation. IL-10 is a key cytokine produced by Tregs that potentiates their suppressive capabilities and helps regulate the immune response [34,35]. IL-10 contributes to preserving the structural integrity of the intestinal barrier, reducing inflammation, and fostering the healing of the intestinal epithelium, thereby impeding the entry of bacteria and antigenic materials [36]. TGF-β, Tregs, and IL-10 collaboratively maintain immune system balance and prevent excessive inflammation [37]. This study found that DSS induced the secretion of the pro-inflammatory cytokines TNF-α, IL-6, and IL-1β in mice, consistent with previous research, and increased the release of the anti-inflammatory factors IL-10 and TGF-β. DBK administration significantly reduced the secretion of pro-inflammatory cytokines, highlighting its anti-inflammatory properties.

**Figure 8:**
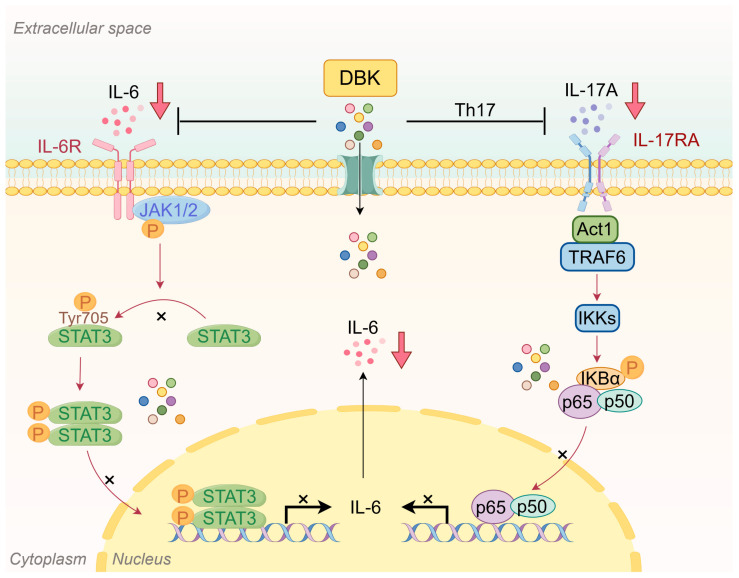
Danggui Beimu Kushen pill (DBK) ameliorates inflammation in colitis mice by modulating the IL-6/IL-6R and IL-17A/IL-17RA signaling pathways (By Figdraw).

The activation of the IL-6/JAK/STAT3 and IL-17/TRAF6/NF-κB pathways induces IL-6 and TNF-α production. IL-6 binds to IL-6R to form a complex that activates the JAK/STAT3 pathway. TNF-α binds to TNF receptors, activating the NF-κB pathway and potentially the JAK/STAT pathway [38]. This positive feedback loop can intensify the inflammatory response, resulting in the increased production of IL-6 and TNF-α, along with an imbalance in other cytokines. DBK can effectively inhibit the positive feedback loop, thus playing a balancing role in immune cells and cytokines (Figure 8).

Despite these interesting findings, the limitations of this study must be highlighted. First, our study used UPLC-ESI-MS/MS to analyze DBK’s complex herbal composition of DBK but did not isolate or test individual components. Although network pharmacology suggests synergistic effects of multiple ingredients, including Angelica sinensis, the exact contributions of each component remain undetermined. We identified potential targets and pathways, such as IL-6/IL-6R and IL-17A/IL-17RA, but these require experimental validation. Future research should dissect individual component interactions within DBK to identify active compounds and address the key limitations of this study. Second, our molecular docking studies are useful for initial screening, but lack experimental validation, necessitating further confirmatory studies. We did not explore potential protein conformational changes induced by small-molecule interactions, which is crucial for understanding regulatory mechanisms. For example, the effect of sitosterol on TGFB1’s conformation and receptor affinity remains uninvestigated. Future research should integrate computational predictions with experimental techniques, such as crystallography and NMR, to validate these interactions and provide a deeper understanding of their molecular mechanism.

## 4. Materials and Methods

### 4.1. Chemical and Reagents

*Angelica sinensis* (Oliv.) Diels (Danggui, Lot.221101), *Fritillaria thunbergii* Miq. (Beimu, Lot.220701), and *Sophora flavescens* Aiton (Kushen, Lot.221001) were obtained from The Second Affiliated Hospital of Anhui University of Chinese Medicine. DSS (Mw40000) was bought from MACLIN (Shanghai, China). Mesalamine (5-ASA, Lot.231215) was purchased from Sunflower Pharmaceutical Group Co., Ltd. (Ha’erbin, China). ELISA kits for detecting IL-23, IL-1β, TNF-α, IL-6, IL-17, TGF-β, and IL-10 in both mouse and human samples were obtained from Jianglai Biology (Shanghai, China). PMA (50 ng/mL), ionomycin (1 µg/mL), and mouse FITC-CD4, PE-IL-17A, and Alexa Fluor 647-FOXP3 antibodies were obtained from BD Biosciences (San Jose, CA, USA). Antibodies against p-NF-κB p65 (3033T) and NF-κB p65 (8242T) were purchased from CST (Danvers, MA, USA). Primary antibodies against IL-1β (ab254360) and TRAF6 (EP591Y) were obtained from Abcam (Cambridge, UK). Antibodies against p-STAT3 (ET1603-40), STAT3 (ET1607-38), p-IκB (ET1609-78), and IκB (ET1603-6) were purchased from HUABIO (Hangzhou, China). The dilutions of the antibodies are shown in Table S2.

### 4.2. DBK Preparation

Danggui, Beimu, and Kushen (1:1:1) were chopped into small pieces and subjected to three extractions with 95% ethanol and two extractions with water, all under reflux at 60 °C. The combined extract was freeze-dried into a powder to produce the total extract (DBK), which was subsequently stored at 4 °C.

### 4.3. UPLC-MS/MS Analysis of the Chemical Constituents of DBK

#### 4.3.1. Dry Sample Extraction

DBK (50 mg) was measured using an electronic balance (MS105DΜ) and combined with 1200 μL of a −20 °C pre-cooled 70% methanolic aqueous internal standard extract. The mixture was vortexed for 30 s every 30 min, repeating this process six times. Following centrifugation at 12,000 rpm for 3 min, the supernatant was removed, filtered through a 0.22 μm microporous membrane, and stored in an injection vial for UPLC-MS/MS analysis.

#### 4.3.2. UPLC Conditions

The extracted samples were examined using a UPLC-ESI-MS/MS setup (ExionLC™ AD) and a tandem mass spectrometry system. An Agilent SB-C18 UPLC column (1.8 µm, 2.1 mm × 100 mm) was employed for the analysis. The mobile phases consisted of solvent A (water with 0.1% formic acid) and solvent B (acetonitrile with 0.1% formic acid). The gradient elution began with 95% A and 5% B, transitioning to a 5% A and 95% B mixture over a 9-minute period, which was then held for an extra minute. The composition of 95% A and 5% B was adjusted within 1.1 min and sustained for 2.9 min. The analysis parameters included a flow rate of 0.35 mL/min, a column temperature of 40 °C, and a 2 μL injection volume. The column output was directed to the ESI-QTRAP-MS system for detection.

#### 4.3.3. ESI-Q TRAP-MS/MS

The ESI source was operated at 500 °C with an ion spray voltage of 5500 V in positive ion mode and −4500 V in negative ion mode. The gasses for ion sources I and II were adjusted to 50 and 60 psi, respectively, and the curtain gas was maintained at 25 psi. Collision-induced dissociation was enhanced to a high setting. Triple quadrupole (QQQ) scans were performed for multiple reaction monitoring (MRM) assays using nitrogen as the collision gas in a medium setting. The declustering potential (DP) and collision energy (CE) of each MRM transition were fine-tuned. MRM transitions were tracked throughout each interval in accordance with the metabolites being eluted.

### 4.4. Animals and Treatments

Male C57BL/6 mice, aged 6–8 weeks and maintained at the SPF level, were sourced from Ziyuan Experimental Animal Technology Co., Ltd., Hangzhou, China. The Animal Ethics Committee of the Anhui University of Chinese Medicine approved all animal studies (Approval No. AHUCM-mouse 2024026, 18 March 2024). Mice were allocated into six random groups: normal, 3% DSS, 5-ASA (300 mg/kg), and DBK at doses of 50, 100, and 200 mg/kg. The drugs were dissolved in saline on the day of administration and administered orally for one week. The daily monitoring of body weight and assessment of fur condition, activity level, and stool blood was conducted using the disease activity index (DAI) score. On the 8th day, the mice were euthanized for blood and colon collection for further analysis.

### 4.5. Routine Blood Test

Blood samples were collected in sodium heparin tubes, gently mixed, and analyzed for lymphocyte (LYMPH), neutrophil (Neu), white blood cell (WBC), and red blood cell (RBC) counts using a Sysmex XN-1000V automated biochemical analyzer (Sysmex Corporation, Norderstedt, Germany).

### 4.6. ELISA Assay

Blood samples were centrifuged at 3000 rpm for 30 min to separate the serum. ELISA kits were used to measure the concentrations of the inflammatory factors IL-23, IL-17, TGF-β, IL-6, TNF-α, IL-10, and IL-1β in mouse serum and colon tissue. All steps were performed according to the manufacturer’s specifications.

### 4.7. H&E, IHC, and IF Staining

H&E, IHC, and IF staining were performed according to the manufacturer’s protocols. The slides were de-paraffinized and rehydrated using xylene, followed by a series of ethanol solutions (100%, 95%, and 75%). IHC and IF staining were performed using primary antibodies targeting p-STAT3 and p-NF-κB p65, respectively. A secondary goat anti-rabbit IgG H&L antibody conjugated with Alexa Fluor 488 was used for immunofluorescence staining. Cell nuclei were stained with DAPI. H&E and IHC slides were imaged using a slide scanner (WISLEAP WS-10, Zhiyue Medical Technology, Changzhou, China). Images were captured with a Leica Stellaris 5 confocal laser-scanning microscope (Leica Microsystems, Wetzlar, Germany).

### 4.8. Analysis of Network Pharmacology

#### 4.8.1. Screening of Active Compounds of DBK and Target Prediction Based on Database

The Traditional Chinese Medicine Systems Pharmacology Database and Analysis Platform [39] (TCMSP) provided the active compounds for Danggui, Beimu, and Kushen. The active compounds of DBK were selected for further analysis based on an oral bioavailability (OB) of at least 30% and a drug-likeness (DL) of at least 0.18. The SDF structures of these compounds were sourced from PubChem and imported into Swiss Target Prediction [40,41] to identify prospective targets.

#### 4.8.2. Construction of the “DBK–Ingredient–Target–UC” Network

To characterize the multi-component treatment characteristics of the three herbal medicines and explore their molecular mechanisms at a systemic level, disease targets for ulcerative colitis were obtained by searching the GeneCards database with “ulcerative colitis” as the keyword. The DBK targets were entered into Cytoscape 3.8.0 to construct a “drug–ingredient–disease–target” network diagram.

#### 4.8.3. Core Targets and KEGG Pathway Enrichment of DBK Against UC

UC-related targets were identified by searching the GeneCard database [42] for the keyword “ulcerative colitis”. We compared the targets of DBK pharmacological compounds with those identified as therapeutic targets for UC. The core targets were then obtained by calculating the topological feature value. Key signaling pathways related to the target genes were identified using KEGG enrichment analysis using the Metascape platform (https://metascape.org/gp/index.html#/main/step1 accessed on 1 December 2024) [43].

### 4.9. Molecular Docking

The atomic structures of IL6, STAT3, TGFB1, and TNF were obtained from the Protein Data Bank and identified using PDB identifiers 7NXZ, 6NJS, 5VQP, and 7JRA, respectively. In the absence of known binding sites for IL6 and TGFB1, SiteMap was used to predict potential docking sites. The Glide application (part of the Schrödinger Suite, version 12.8) was used for docking experiments. Protein models were refined with the Protein Preparation Wizard by applying the OPLS4 force field prior to docking. This process includes reassigning bond orders, adding hydrogens, eliminating water molecules and co-crystallized ions, and reassigning hydrogen bonds with PROPKA. The refined structures were then subjected to energy minimization using the OPLS4 force field at its standard parameters. Ligand structures were drawn with a 2D Sketcher and refined with LigPrep, adhering to default parameters. A docking grid was defined based on the identified ligand-binding sites. The docking process was performed using the extra precision (XP) scoring method. The PyMol tool was used to visualize the complexes with the lowest binding energies.

### 4.10. RNA Extraction and Quantitative Real-Time PCR (qRT-PCR)

Total RNA was extracted from colon tissues using an RNA extraction kit (SparkJade, China), and first-strand cDNA was synthesized using a reverse transcription kit from the same manufacturer. SYBR Green (YEASEN, Shanghai, China) was utilized for real-time quantitative PCR. Supplementary Table S3 lists the primer sequences used. β-actin mRNA level was used as an endogenous control for the target gene.

### 4.11. Western Blotting

Tissue samples from the colon were processed using a tissue homogenizer in RIPA buffer enhanced with 1 mM inhibitors of proteases and phosphoproteinases. Protein content was quantified using a BCA assay kit (SparkJade, Jinan, China). The proteins were then separated by 12% SDS-PAGE and electrophoretically transferred onto PVDF membranes. Following this, the membranes were exposed to primary antibodies for overnight incubation at 4 °C. Protein bands were detected using enhanced chemiluminescence reagents (Abbkine, Wuhan, China) and captured on a Tanon-5200 Chemiluminescent Imaging System (Tanon, Shanghai, China).

### 4.12. Flow Cytometry

The spleens and MLNs were gently ground in a Petri dish with 1 mL of PBS on ice. The cell suspension was collected and filtrated through a 70 μm cell sieve to remove tissue residue. The supernatant was discarded following centrifugation at 350× *g* for 10 min. The cells underwent red blood cell lysis, were incubated for 15 min, and then centrifuged at 350× *g* for 5 min to eliminate hemocytes. The cells were then washed twice with 1 mL of staining buffer consisting of PBS with 3% FBS. The cells were cultured in RPMI 1640 medium supplemented with 1% penicillin–streptomycin, 5% fetal bovine serum, 50 ng/mL PMA, and 1 µg/mL ionomycin. The cells were incubated at 37 °C in a CO_2_ incubator for 4 h, followed by another washing step. Then, the cells were stained with an appropriate amount of cell surface staining antibodies (CD4) in the dark (30 min, 4 °C). Subsequently, the cells were diligently resuspended and subjected to fixation and permeabilization by incorporating 1 mL of a fixative/permeabilization reagent per sample for a 20-min duration at 4 °C. This step was succeeded by two rinses with Perm/Wash buffer, which was subsequently removed by centrifugation at 300× *g* for 5 min. Next, the cells were kept in the dark at 4 °C for 40 min while being treated with antibodies specific to Foxp3 and IL-17A for intracellular staining. After washing, the cells were reconstituted in 500 μL of staining buffer and examined by flow cytometry. The acquired data were then processed and depicted using FlowJo 10.8.1.

### 4.13. Statistical Analysis

Results are expressed as mean ± standard deviation (SD) from a minimum of three independent experiments and analyzed using one-way ANOVA with SPSS Statistics 26.0 and GraphPad Prism 8. The significance levels are indicated as follows: * *p* < 0.05; ** *p* < 0.01; and *** *p* < 0.001.

## 5. Conclusions

In conclusion, DBK successfully reduced inflammation in DSS-induced UC mice by modulating the IL-6/IL-6R and IL-17A/IL-17RA pathways, as evidenced by alleviated colitis symptoms, decreased pro-inflammatory cytokines (TNF-α, IL-6, IL-1β), and histological improvements. The findings presented here in not only validate the efficacy of DBK but also offer novel perspectives on its application. Furthermore, this study provides innovative insights into the treatment and prevention strategies for UC, potentially laying the groundwork for the development of more targeted and effective therapeutic interventions based on traditional Chinese pharmacological principles.

## Figures and Tables

**Table 1:** Five key components of DBK were identified using a UPLC-ESI-MS/MS system.

Compounds	CAS	Formula	Q1 (Da)	Q3 (Da)	Molecular Weight (Da)	Ionization Model	Relative Peak Areas
Ferulic acid	537-98-4	C_10_H_10_O_4_	193.05	134.04	194.0579	[M-H]-	187,046,678
Matrine	519-02-8	C_15_H_24_N_2_O	249.2	148.11	248.1889	[M+H]+	371,862,529
Oxymatrine	16837-52-8	C_15_H_24_N_2_O_2_	265.1911	247.1819	264.1838	[M+H]+	294,617,063
Verticine	23496-41-5	C_27_H_45_NO_3_	432.35	414.34	431.3399	[M+H]+	231,012,622
Peiminine	18059-10-4	C_27_H_43_NO_3_	430.33	412.33	429.3243	[M+H]+	219,523,435

## Data Availability

Data are contained within the article.

## Supplementary Materials

The following supporting information can be downloaded at: https://www.mdpi.com/article/10.3390/ph18020141/s1, Table S1: Active ingredients of DBK; Table S2: The dilutions of the antibodies; Table S3: Primers used in the quantity real-time PCR assay.
