# Supplementary material for: Danggui Beimu Kushen Pill Alleviates Colitis-Induced Inflammation in Mice by Regulating the IL-6/IL-6R and IL-17A/IL-17RA Signaling Pathways
Source: Pharmaceuticals (Basel). 2025 Jan 22;18(2):141. doi: 10.3390/ph18020141 (PMC11858545; doi:10.3390/ph18020141)
Supplement: Supplementary file 1 [file pharmaceuticals-18-00141-s001.zip › Table S1 Active ingredients of DBK.pdf]

Table S1. Active ingredients of DBK.

No.	Molecule Name	Origin
A	Sitosterol	*Angelica sinensis* (Oliv.) Diels (Dang Gui); *Fritillaria thunbergii* Miq. (Bei Mu)
DG2	Myrcene	*Angelica sinensis* (Oliv.) Diels (Dang Gui)
DG3	Levistolide A	*Angelica sinensis* (Oliv.) Diels (Dang Gui)
DG4	Succinic acid	*Angelica sinensis* (Oliv.) Diels (Dang Gui)
DG5	Cadinene	*Angelica sinensis* (Oliv.) Diels (Dang Gui)
DG6	Scopoletol	*Angelica sinensis* (Oliv.) Diels (Dang Gui)
DG7	Nicotinic acid	*Angelica sinensis* (Oliv.) Diels (Dang Gui)
DG8	Vanillin	*Angelica sinensis* (Oliv.) Diels (Dang Gui)
DG9	Palmitic acid	*Angelica sinensis* (Oliv.) Diels (Dang Gui)
DG10	Senkyunolide	*Angelica sinensis* (Oliv.) Diels (Dang Gui)
BM1	Pelargonidin	*Fritillaria thunbergii* Miq. (Bei Mu)
BM3	Peimisine	*Fritillaria thunbergii* Miq. (Bei Mu)
BM4	Zhebeiresinol	*Fritillaria thunbergii* Miq. (Bei Mu)
BM5	Ziebeimine	*Fritillaria thunbergii* Miq. (Bei Mu)
BM6	Syringaresinol	*Fritillaria thunbergii* Miq. (Bei Mu)
BM7	Peiminine	*Fritillaria thunbergii* Miq. (Bei Mu)
KS1	Naringenin	*Sophora flavescens* Aiton (Ku Shen)
KS2	Inermine	*Sophora flavescens* Aiton (Ku Shen)
KS3	8-Isopentenyl-kaempferol	*Sophora flavescens* Aiton (Ku Shen)
KS4	Inermin	*Sophora flavescens* Aiton (Ku Shen)
KS5	Wighteone	*Sophora flavescens* Aiton (Ku Shen)
KS6	Sophoramine	*Sophora flavescens* Aiton (Ku Shen)
KS7	Sophoridine	*Sophora flavescens* Aiton (Ku Shen)
KS8	Formononetin	*Sophora flavescens* Aiton (Ku Shen)
KS9	cis-Dihydroquercetin	*Sophora flavescens* Aiton (Ku Shen)
KS10	Liquiritigenin	*Sophora flavescens* Aiton (Ku Shen)
KS11	rac-Hesperetin	*Sophora flavescens* Aiton (Ku Shen)
KS12	Matrine	*Sophora flavescens* Aiton (Ku Shen)
KS13	Luteolin	*Sophora flavescens* Aiton (Ku Shen)
KS14	(+)-14alpha-hydroxymatrine	*Sophora flavescens* Aiton (Ku Shen)
KS15	Lemannine	*Sophora flavescens* Aiton (Ku Shen)
KS16	Anagyrine	*Sophora flavescens* Aiton (Ku Shen)
KS17	5,9-Dihydroxymatrine	*Sophora flavescens* Aiton (Ku Shen)
KS18	Glyceollin	*Sophora flavescens* Aiton (Ku Shen)
KS19	Hyperforin	*Sophora flavescens* Aiton (Ku Shen)
KS20	Kushenin	*Sophora flavescens* Aiton (Ku Shen)

(Continued)

No.	Molecule Name	Traditional Chinese Medicine
KS21	Leachianone G	*Sophora flavescens* Aiton (Ku Shen)
KS22	Lehmanine	*Sophora flavescens* Aiton (Ku Shen)
KS23	Lupanine	*Sophora flavescens* Aiton (Ku Shen)
KS24	Norartocarpetin	*Sophora flavescens* Aiton (Ku Shen)
KS25	Phaseolin	*Sophora flavescens* Aiton (Ku Shen)
KS26	Sophranol	*Sophora flavescens* Aiton (Ku Shen)
KS27	Quercetin	*Sophora flavescens* Aiton (Ku Shen)
