# Supplementary material for: Danggui Beimu Kushen Pill Alleviates Colitis-Induced Inflammation in Mice by Regulating the IL-6/IL-6R and IL-17A/IL-17RA Signaling Pathways
Source: Pharmaceuticals (Basel). 2025 Jan 22;18(2):141. doi: 10.3390/ph18020141 (PMC11858545; doi:10.3390/ph18020141)
Supplement: Supplementary file 1 [file pharmaceuticals-18-00141-s001.zip › Table S2 The dilutions of the antibodies.pdf]

Table S2. The dilutions of the antibodies.

Primary antibody	Company	Dilution	Lot
Anti-phospho-STAT3(Tyr705)	HUABIO	1:5000（WB）<br>1:200（IHC）<br>1:200（IF）	ET1607-38
Anti-STAT3	HUABIO	1:1000（WB）	ET1603-40
IL-6	HUABIO	1:1000（WB）	R1412-2
IL-6R	MCE	1:1000（WB）	4899969
IL-17RA	Affinity Biosciences	1:1000（WB）	DF3602
IL-17A	Affinity Biosciences	1:1000（WB）	DF6127
TRAF6	AbcamTechnology	1:1000（WB）	EP591Y
Anti-phospho--IκB	HUABIO	1:1000（WB）	ET1609-78
Anti-IκB	HUABIO	1:1000（WB）	ET1603-6
Phospho-NF-kB p65	Cell Signaling Technology	1:1000（WB）<br>1:200（IF）	3033s
NF-κB p65	Cell Signaling Technology	1:1000（WB）	8242T
Anti-beta Actin Antibody	HUABIO	1:10000（WB）	PSH03-63
Goat Anti-Rabbit IgG H&L(HRP)	ZEN-BIOSCIENCE	1:10000（WB）	511203
H3	Cell Signaling Technology	1:2000（WB）	4499T
Goat Anti-Rabbit IgG H&L (AF488)	ZEN-BIOSCIENCE	1:2000（IF）	550037
