# Supplementary material for: Danggui Beimu Kushen Pill Alleviates Colitis-Induced Inflammation in Mice by Regulating the IL-6/IL-6R and IL-17A/IL-17RA Signaling Pathways
Source: Pharmaceuticals (Basel). 2025 Jan 22;18(2):141. doi: 10.3390/ph18020141 (PMC11858545; doi:10.3390/ph18020141)
Supplement: Supplementary file 1 [file pharmaceuticals-18-00141-s001.zip › Table S3 Primers used in the quantity real-time PCR assay.pdf]

Table S3. Primers used in the quantity real-time PCR assay.

Gene	FORWARD	REVERSE
IL-6	GTCAGGTGGAGATCTCTTCTTG	GTCACCAGCATCAGTCCCAAGAAG
FOXP3	AAGAATGCCATCCGCCACAAC	TCATCTACGGTCCACACTGCTC
IL-17RA	GAGCCGACAGAAGCAGGAGATG	CTTCCAGTGGTCACACCGTAGC
TRAF6	GCAACTCTTACAGCCAGGAAACTTC	ACCACTGAGCCAATTCTCCAACC
ROR-γt	GTCCCGAGATGCTGTCAAGTTTG	TTCCTGTTGCTGCTGCTGTTG
IL-17A	AGGCAGCAGCGATCATCCC	TGGAACGGTTGAGGTAGTCTGAG
TNF-α	AAGACACCATGAGCACAGAAAGC	GCCACAAGCAGGAATGAGAAGAG
IL-10	GGACAACATACTGCTAACCGACTC	GATTTCTGGGCCATGCTTCTCTG
IL-1β	CACCTCACAAGCAGAGCACAAG	GCATTAGAAACAGTCCAGCCCATAC
β-actin	ACTGCCGCATCCTCTTCCTC	AACCGCTCGTTGCCAATAGTG
